# Does an Over-Connected Visual Cortex Undermine Efforts to Stay Sober After Treatment for Alcohol Use Disorder?

**DOI:** 10.3389/fpsyt.2020.536706

**Published:** 2020-12-10

**Authors:** Angela M. Muller, Dieter J. Meyerhoff

**Affiliations:** Department of Radiology and Biomedical Imaging, University of California, San Francisco, San Francisco, CA, United States

**Keywords:** segregation, integration, modules, provincial hub, intrinsic, alcohol relapse marker, default mode network, participation coefficient

## Abstract

A fine-tuned interplay of highly synchronized activity within and between the brain's communities is a crucial feature of the brain's functional organization. We wanted to investigate in individuals with alcohol use disorder (AUD) the degree to which the interplay of the brain's community-architecture and the extended brain reward system (eBRS) is affected by drinking status (relapse or abstinence). We used Graph Theory Analysis of resting-state fMRI data from treatment seekers at 1 month of abstinence to model the brain's intrinsic community configuration and their follow-up data as abstainers or relapsers 3 months later to quantify the degree of global across-community interaction between the eBRS and the intrinsic communities at both timepoints. After 1 month of abstinence, the ventromedial PFC in particular showed a significantly higher global across-community interaction in the 22 future relapsers when compared to 30 light/non-drinking controls. These differences were no longer present 3 months later when the relapsers had resumed drinking. We found no significant differences between abstainers and controls at either timepoint. *Post hoc* tests revealed that one eBRS region, the ventromedial PFC, showed a significant global across-community interaction with a community comprising the visual cortex in relapsers at baseline. In contrast, abstainers showed a significant negative association of the ventromedial PFC with the visual cortex. The increased across-community interaction of the ventromedial PFC and the visual cortex in relapsers at timepoint 1 may be an early indicator for treatment failure in a subgroup of AUD patients.

## Introduction

The brain is a complex functional network that is hierarchically organized in multiple levels of smaller subnetworks or communities nested within each other. A crucial feature of the brain's community organization - and an important requirement for efficient processing - is that the regions belonging to the same community are densely inter-connected, whereas the connections between the communities are sparse (1, 2). As a result, just a few distinct brain regions are engaged in information integration across the communities, which are otherwise functionally segregated (1–3).

This fine-tuned interplay between integration and segregation is a hallmark of the brain's functional organization when engaged in goal-driven behavior or cognitive tasks (4–6) and at rest (3, 7, 8). Integration and segregation are two opposite positions on a continuum of the brain's modus operandi. Any shift toward an extreme of the continuum is disturbing this fine-tuned balance leading to either over-integration, or over-segregation. Both over-integration and over-segregation have been shown to have significant effects on cognition and behavior in healthy populations (3, 9–12), but this concept also helps to understand psychiatric and neurological conditions like traumatic brain injury (13), schizophrenia (14), Alzheimer's disease (15, 16), and attention deficit/hyperactivity disorder (17).

Neurotransmitters like glutamate and ρ-aminobutyric acid (GABA) are intimately involved in orchestrating the excitatory/inhibitory interplay that shapes this within-community and between-community architecture of the brain (10, 12, 18–25). Chronic alcohol exposure and abstinence during treatment for alcohol use disorder (AUD) have been shown to result in neuroadaptations in GABAergic and glutamatergic synaptic transmission, especially in the brain reward system (26–31). These AUD related neuroadaptations have also consequences for the future course of the alcohol dependence (32–34).

Since research has shown an altered inhibitory and excitatory neurotransmitter tone in AUD individuals during the different stages of a typical alcohol use cycle (active use, withdrawal, remission), we reason that we might gain new insights into the effects of AUD on the brain by investigating the intrinsic community architecture in treatment seekers and how this community architecture relates to the extended brain reward system [eBRS, (35, 36)] and treatment outcome. To that aim, we modeled the brain's community organization and the degree of interaction between the communities in patients at 1 month of abstinence and 4 months into treatment when they were either abstinent or had resumed heavy drinking. We had two main hypotheses: 1. the interaction of the eBRS regions within its own community is differentially altered in relapsers (REL) and abstainers (ABS) when compared with healthy controls (CON), and 2. the interaction of the eBRS regions with the other communities differs between relapsers and abstainers when compared with controls.

## Methods

### Participants

Included in this analysis were the resting-state functional MRI (rs-fMRI) data of 34 AUD individuals (mean age 41.7 of years; SD 9.9) obtained within 4 weeks of abstinence from alcohol while in outpatient treatment [timepoint 1 (TP1)]. They had been recruited from the San Francisco Veterans Administration Medical Center Substance Abuse Day Hospital and the Kaiser Permanente Chemical Dependence Recovery outpatient treatment clinics. Twenty-four of these AUD individuals (mean age 41.1 of years, SD 9.0) returned to undergo the same research protocol three months later (TP2). Demographics and clinical characteristics are given in Table 1a (baseline), and Table 1b (follow-up). Drinking status (abstinent or relapsed) of the AUD individuals at timepoint 2 determined their group membership for the purpose of this analysis. Twenty-two abstinent AUD individuals studied at timepoint 1 were future relapsers (REL), 14 of whom returned for the 3 month-follow-up; the other 12 AUD individuals were abstinent at both timepoints (ABS), and 10 of them returned for follow-up. Thirty light/non-drinkers were recruited as control participants from the local community (CON), 21 of whom came back for a follow-up. Some demographic data given in Table 1a was not available for all control participants since controls were recruited for three different but contemporaneous research studies that acquired slightly different demographic information.

**Table 1a:** Demographic description of the three groups: Controls (CON), Abstainers (ABS), Relapsers (REL).

	**TP1**			**TP2**		
	**CON**	**ABS**	**REL**	**CON**	**ABS**	**REL**
**N**	**30**	**12**	**22**	**21**	**10**	**14**
Age [years]	44.2 (11.1)	37.1 (7.8)	43.9 (10.4)	42.6 (10.9)	38.1 (8.5)	43.2 (9.2)
Gender F|M [n|	12|18	8|4	8|14	7|14	6|4	5|9
Education [years]	15.9 (2.4)	14.7 (1.9)	14.8 (2.0)	16.1 (2.5)	14.7 (1.6)	15.6 (1.6)
1-year average drinks/month	6.1 (8.3)	367.0 (206.1)**	355.2 (243.8)**	7.5 (9.9)	329.6 (192.9)*	384.8 (297.4)**
Lifetime average drinks/month	8.7 (7.3)	183.9 (93.6)**	190.4 (98.6)**	7.9 (6.8)	160.9 (80.7)**	197.6 (111.8)*
Smokers no | current | former [n]	15|3|4	7|2|3	10|9|3	8|3|2	6|2|2	7|4|3
FTND total score	2.1 (2.0)	1.6 (2.3)	2.7 (2.1)	2.0 (1.8)	1.3 (1.5)	2.3 (2.2)
BIS-II-total score	55.1 (9.9)	65.8 (8.8)*	66.6 (11.7)**	54.4 (10.9)	62.3 (11.6)	63.1 (9.3)*
BDI_total score	3.1 (3.9)	12.0 (8.9)**	15.0 (7.5)**	2.6 (2.8)	7 (6.8)	9.9 (5.8)**
STAI_state score	23.9 (5.4)	37.5 (11.0)*	38.2 (12.6)**	25.3 (8.8)	35.7 (12.0)*	33.5 (9.6)
STAI_trait score	30.9 (8.9)	47.0 (11.7)**	47.5 (11.5)**	30.1 (6.8)	41.9 (10.6)*	38.8 (10.5)*

*Mean value with SD in brackets; one asterisk = p < 0.05, two asterisks = p < 0.001 indicate the statistical level on which the two AUD groups differed from the controls as the reference group. The controls were pooled from three contemporaneously performed research studies, so not all demographic variables were consistently available. The variables other than age, gender and education were obtained from 22 controls at timepoint 1 and 9 controls at timepoint 2. Abbreviations: TP, timepoint; FTND, Fagerstrom Test for Nicotine Dependence; BIS-II, Barratt Impulsiveness Scale; BDI, Beck Depression Inventory; STAI State/STAI Trait, State-Trait Anxiety Inventory State/Trait*.

**Table 1b:** Comparison of participants with and without follow-up.

	**AUD at TP1**		**ABS at TP1**		**REL at TP1**	
	**With follow-up**	**Dropouts**	**With follow-up**	**Dropouts**	**With follow up**	**Dropouts**
**N**	**24**	**10**	**10**	**2**	**14**	**8**
Age [years]	40.8 (8.9)	43.9 (12.1)	37.9 (8.4)	36.3 (5.5)	42.9 (9.1)	45.9 (12.7)
Gender F|M [n]	11|13	5|5	6|4	2|0	5|9	3|5
Education [years]	15.3 (1.8)	13.6 (1.9)*	15.1 (1.9)	13.0 (1.4)	15.5 (1.8)	13.8 (2.1)*
1-year average drinks/month	361.8 (255.6)	353.6 (154.1)	329.6 (192.9)	554.2 (220.3)	384.8 (297.5)	303.4 (96.1)
Lifetime average drinks/months	182.3 (99.79)	202.1 (87.4)	160.9 (80.7)	298.9 (77.5)*	197.6 (111.8)	177.9 (75.1)
Smokers no | current | former [n]	13|6|5	4|5|1	6|1|3	1|1|0	7|5|2	3|4|1
FTND total score	1.8 (2.0)	3.3 (2.1)	5	0.7 (1.5)*	2.4 (2.1)	3.0 (2.1)
BIS-II-total score	66.4 (10.6)	66.3 (11.4)	67.2 (9.2)	59.0 (1.4)	65.8 (11.9)	68.1 (12.1)
BDI_total score	14.4 (6.8)	12.9 (8.6)*	13.4 (9.1)	5.0 (2.8)	15.1 (8.4)	14.9 (6.0)
STAI_state score	37.4 (10.6)	39.4 (15.2)	38.8 (11.43)	31.0 (7.1)	36.4 (10.2)	41.5 (16.3)
STAI_trait score	46.9 (11.7)	48.2 (11.1)*	47.6 (12.7)	44.0 (4.2)	46.4 (11.4)	49.2 (12.2)*

*Mean value with standard deviation in brackets; an asterisk indicates significant differences between dropouts vs. participants with follow up at p < 0.05. Abbreviations: TP, timepoint; FTND, Fagerstrom Test for Nicotine Dependence; BIS-II, Barratt Impulsiveness Scale; BDI, Beck Depression Inventory; STAI State/STAI Trait, State-Trait Anxiety Inventory State/Trait*.

All study participants were administered the screening section of the Structural Clinical Interview for DSM-5 Axis I disorders. All AUD individuals had moderate or severe AUD and no other moderate or severe substance use disorder. Exclusion criteria for all participants included a history of neurologic disorder, e.g., epilepsy, traumatic brain injury with loss of consciences > 30 min, cerebrovascular disease, a history of general medical disease such as untreated hypertension, diabetes, hypo/hyperthyroidism, and of psychiatric diseases such as major depression, anxiety, trauma. In addition, all study participants had to have at least 5 min of clean rs-fMRI data left after cleaning the data from motion and physiological noise.

All participants were also assessed by a battery of interviews and standardized questionnaires that included the Beck Depression Inventory [BDI; (37)], Barratt Impulsiveness Scale [BIS; (38)], as well as standardized questionnaires assessing lifetime substance use (alcohol and other substances including tobacco). AUD individuals had a history of consuming at least 80 standard alcoholic drinks per months (>150 for men) for > 6 years (> 8 years in men) before treatment. Controls had consumed fewer than 60 standard alcoholic drinks in any month over lifetime (1 standard alcoholic drink contains 13.6 g of ethanol). The committees of human research at the University of California San Francisco and the VA Medical Center had approved the study and informed consent was obtained from each participant prior to any research procedures in accordance with the Declaration of Helsinki.

### MRI Data

The MRI data were collected at the VA Medical Center San Francisco on a 3.0 T MRI scanner (Siemens Magnetom Skyra Syngo MR D13) using a 20 channel receive head coil. The study protocol included different types of structural imaging, as well as rs-fMRI. For this study were used: (a) A T1 weighted MPRAGE sequence with repetition time (TR) = 2,300 ms, echo time (TE) = 2.98 ms, flip angle 9^0^, field of view (FOV) 192 × 256 × 256 mm^3^, isotropic voxel size 1 × 1 × 1 mm^3^, 256 slices per volume, acquisition duration = 5.28 min. (b) A T2 weighted TSE sequence with TR = 3,210 ms, TE = 11 ms, flip angle = 150^0^, FOV = 230 × 230 × 54 mm^3^, anisotropic voxel size 0.9 × 0.9 × 3 mm^3^, 54 slices per volume, acquisition duration = 3.21 min. (c) A whole brain task-free echo planar imaging blood oxygen level dependent echo-planar 2D PACE sequence with TR = 2,020 ms, TE = 27 ms, 37 transverse slices (FOV = 1,320 × 1,320 mm^2^; Matrix size 88 × 88, descending acquisition without a gap, anisotropic voxel size 2.5 × 2.5 × 3.5 mm^3^), 240 volumes and 8 min acquisition duration.

#### MRI Pre-processing

General pre-processing: All pre-processing steps of the MRI data were performed using SPM12 (https://www.fil.ion.ucl.ac.uk/spm/) running on MATLAB 2018b. First, the first ten volumes of the echo planar imaging (EPI) data were discarded reducing T1 saturation effects, leaving 230 volumes for analysis. Then the EPI data were slice time-corrected for descending acquisition and a first alignment of the EPI data was performed by using a two-pass procedure where the EPI images were first aligned to the first image of the series and then again aligned to the mean EPI image of the first run. Only the motion-parameter file and the mean image of that first alignment step were used for the further pre-processing steps. Next, the T2 weighted image was co-registered to the T1 weighted image and afterwards the mean EPI image co-registered to the T2 weighted image, whereby it was also indirectly co-registering the EPI image to the T1 weighted image. A second alignment step was then performed by which the original 230 EPI volumes were co-registered to the mean EPI image (now co-registered to the T1 weighted image) from the first alignment. Next, the T1 weighted image was segmented into gray, white and CSF tissue maps using the “New Segmentation” algorithm of SPM12. The DARTEL procedure (39) was run to create a study population specific template that was used for the subsequent normalization step in which the structural and functional images were normalized to the MNI-space. During this step the functional and structural images were resampled to a 2 × 2 × 2 mm^3^ isotropic voxel size and only minimally smoothed using an isotropic Gaussian kernel (FWHM 1 mm) in order to minimize possible spurious correlations for the subsequent graph theoretical analyses (40).

#### Denoising of the rs-fMRI Data and Computing Correlation Matrices

Conn (Version18b, https://www.nitrc.org/projects/conn/) running on MATLAB (MATLAB 2018b) was used to denoise the rs-fMRI data and to compute participant-specific Pearson correlation matrices. The Artifact Detection Toolbox (ART) as implemented in the pre-processing pipeline of Conn was used to identify motion corrupted outliers in the fMRI time-series (global-signal z-value threshold 5; subject-motion mm threshold 0.9). The anatCompCor method (41) was used to detect further low-frequency physiological confounding signals such as heart rate or respiration that can modulate the BOLD signal and influence the connectivity strength. The volumes identified as outliers were then censored by dummy-coding them as nuisance regressors and regressed out together with the confounding signals calculated by the CompCor method. Subsequently, the data were band-pass filtered (0.008–0.09 Hz), detrended (linear, quadratic and cubic), and despiked. We used *t*-tests and the *composite motion measure* implemented in ART and the number of motion corrupted outlier volumes as identified by the denoising procedure to assess that the three groups did not significantly differ in motion artifacts and confounding signals, see Table 2.

**Table 2:** Results of the Student *t*-Tests for Group-Specific Differences in Motion Artifacts and other Physiological Confounds.

	**TP1**			**TP2**		
	**Mean**	**SD**	***p*-value**	**Mean**	**SD**	***p*-value**
**Maximum voxel**						
**displacement in mm**						
CON vs. ABS	0.304/0.254	0.190/0.098	0.38	0.327/0.272	0.181/0.183	0.41
CON vs. REL	0.304/0.346	0.190/0.158	0.37	0.327/0.296	0.181/0.143	0.61
ABS vs. REL	0.254/0.346	0.098/0.158	0.13	0.272/0.296	0.183/0.143	0.73
**Number of motion corrupted outlier volumes**						
CON vs. ABS	17.6/7.4	19.7/8.4	0.11	16.6/9.7	21.0/21.0	0.34
CON vs. REL	17.6/17.3	19.7/20.9	0.95	16.6/9.5	21.0/12.0	0.27
ABS vs. REL	7.4/7.3	8.4/20.9	0.14	9.7/9.2	21.0/12.0	0.98

### Graph Theoretical Analyses (GTA)

To specify nodes for the GTA, we used the Atlas of Intrinsic Connectivity of Homotopic Areas [AICHA, (42)]. This parcellation fulfills two mandatory requirements for GTA because each of its 384 ROIs is characterized by functional homogeneity of the constituting voxels and the parcellation covers the entire cortex and all subcortical structures (42). Since we wanted to investigate the interplay between functional integration and segregation in the brain of participants with AUD, we used both positive and negative BOLD-signal correlations to define the edges between the nodes. Integration corresponds to synchronized brain activity of a group of brain regions measured/operationalized as positive correlations of the BOLD signal between these regions. Segregation as the counterpart of integration corresponds best to anticorrelated BOLD signal activity between regions.

#### Defining the Extended Brain Reward System (eBRS) Using AICHA ROIs

**Figure 1:**
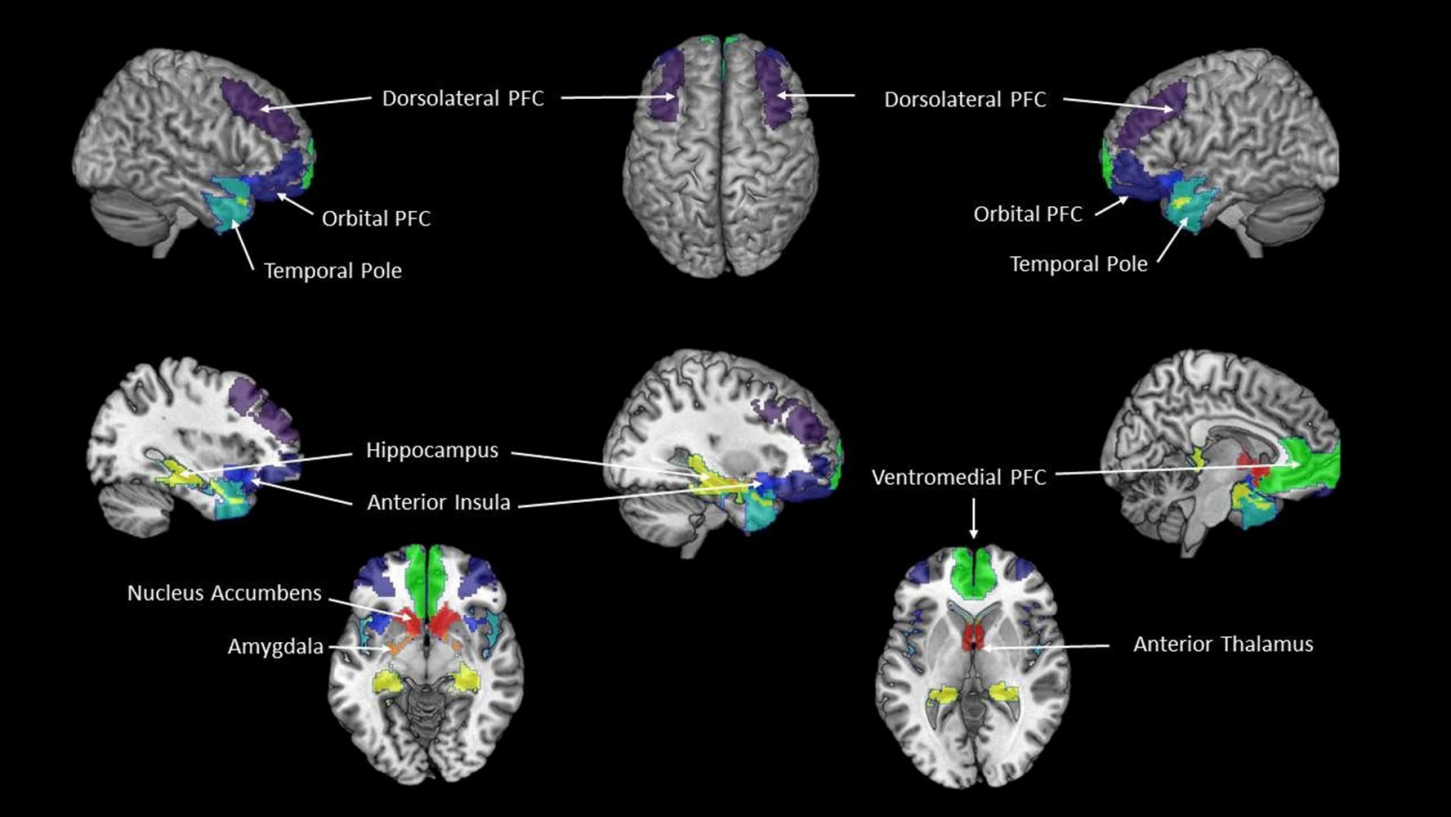
The extended Brain Reward System (eBRS). The nine brain regions of the extended brain reward system–plum = dorsolateral PFC, dark blue = orbital PFC; cyan = temporal pole; pistachio-green = hippocampus; navy-blue = anterior insula; yellow = amygdala; orange-red = nucleus accumbens, dark red = anterior thalamus; lime-green = ventromedial PFC.

Several well-established intrinsic connectivity networks, e.g., the executive-control network or the attention networks, have intrinsic activity patterns that are very similar to those that can be observed with task-based fMRI when the person is actively engaged in one of these activities (43). The eBRS, however, does not have such an intrinsic activity equivalent and in order to study it using rs-fMRI, it must be modeled. Using 62 ROIs of the AICHA parcellation, we built the following composites representing the nine regions of the eBRS (35, 36): left and right nucleus accumbens, anterior thalamus, amygdala, hippocampus and parahippocampal gyrus, anterior insula, lateral orbital prefrontal cortex, dorsolateral prefrontal cortex, ventromedial prefrontal cortex, and temporal pole). To compute region-specific GTA values for our analyses, we averaged the GTA values of the constituent AICHA ROIs of each of the nine eBRS composites (Figure 1).

#### Modeling the eBRS' Over-Integration or Disconnection on Community-Level With GTA

##### First Step: Defining the Group-Specific Community Configuration at Timepoint 1

To address our first hypothesis, we determined the underlying community configuration of for each of three groups at timepoint 1. For that purpose, we used a data-driven approach and ran 10,000 iterations of the community_Louvain algorithm (γ = 1.6 [Brain Connectivity toolbox (BCT), (44)] on each participant's individual connectivity matrix from timepoint 1 while simultaneously computing a participant-specific agreement matrix, which coded how often two nodes were allocated to the same community over the 10,000 iterations. Next, we computed three group-specific average agreement matrices which were then used as input to compute a group-specific consensus partition. A consensus partition allows to find the community structure that represents the community configuration characteristics that were most commonly shared by all participants and across all iterations. To that end, the Louvain algorithm was re-run with 10,000 iterations [Brain Connectivity toolbox (BCT), (44)] on the average agreement matrix of each group until the re-clustered agreement matrices converged into a single cluster solution for that group at timepoint 1.

##### Second Step: Determining the Degree of Global Across-Community Integration of Each of the 384 Nodes

All GTA measurements described in the two next sections were computed for each of the individual 384 nodes of the AICHA parcellation at first and then averaged [1] across the nodes that formed one of the nine regions of the eBRS as defined above and [2] across the nodes that were members of the same community.

The group-specific community configuration at timepoint 1 was used to inform the computation of the participant coefficient (PC) for each participant individually at timepoint 1 (with the participant's connectivity matrix from timepoint 1) and at timepoint 2 (with the participant's connectivity matrix from timepoint 2) according to the participant's group membership.

The PC [Brain Connectivity toolbox (BCT), (44)] measures to which degree a node is involved in the global across-community information integration. A node with a PC (= “positive” PC) value near 1 has a high number of connections with nodes from other communities and qualifies as a connector hub that facilitates the inter-community integration (2). When using signed weighted matrices, i.e., connectivity matrices with positive and negative correlations, it is possible to compute a second PC value (= “negative” PC) for the negatively correlated edges of every individual node as well (45). A node with a “negative” PC value near 1 is highly segregated or even disconnected from the global inter-community information integration. The information of the “positive” PC value and the “negative” PC value can be combined to describe a node's overall importance for the communication between communities. In that context a connector hub is defined by a high “positive” PC and a “negative” PC near zero, whereas a disconnected or separated node is characterized by a high “negative” PC combined with a very low “positive” PC value (45).

To be able to quantify a node's engagement in the between-module information integration, we subtracted the “negative” PC value of each node from its “positive” PC value. A positive result of that subtraction indicates that the inter-community integrative role of that node is relatively higher than its role in separating the communities from each other, while a negative result indicates that the node is mainly segregated from the global inter-community integration. For the remainder of the paper, we call this new measurement we derived from the subtraction of the two PC values “global integration coefficient” or GIC.

In order to differentiate beneficial integration or segregation from maladaptive “over-integration” or “over-segregation,” a reference value was defined, above or below which a node was considered “over-integrated” or “disconnected,” respectively. We used our control participants as reference group. Specifically, we defined that a node showed over-integration whenever the result of the subtraction of the “negative” PC value from the “positive” PC value (i.e., the GIC value) was significantly higher than the reference value of the same node in the controls. Vice versa, a node with a GIC value significantly lower than the same node's reference value in the controls qualified as “disconnected.”

##### Third Step: Determining the Centrality of the eBRS Regions for Within-Community-Crosstalk

Another feature of an efficient network organization is that not all nodes belonging to the same community are equally important for the information integration within that community. Some nodes are more densely connected within their own community than the other nodes in the community to the effect that these nodes are central for the communication within the community. Such nodes are called “provincial hubs.” However, as it is the case with connector hubs controlling the information integration across communities, too many nodes qualifying as provincial hubs within the same community make the network organization inefficient and are also an indicator of (community-intern) over-integration.

We used the “within-module-degree-z-score” algorithm [Brain Connectivity toolbox (BCT), (45)] to determine the eBRS regions' centrality for the information integration within their respective communities. A positive within-community centrality value characterizes a node highly connected with the other nodes of its community, whereas a negative within-community centrality value indicates a node that is mostly segregated from the within-community information integration (2, 44).

#### Statistics

As neither of the two PC values nor the values of the resultant GIC met the assumption for parametric *t*-tests, we used non-parametric Wilcoxon Rank Sum tests to test for significant differences between the three groups (significance level *p* = 0.05). Likewise, we used Spearman's rho to compute GIC associations between the regions of the eBRS and the communities within each group at each timepoint with the aim to determine the communities with whom the regions of the eBRS showed over-integration or segregation. To compute group differences in within-community centrality, we used Student *t*-tests (significance level *p* = 0.05). As we proposed specific hypotheses regarding the eBRS regions crosstalk within their own community and across the communities (i.e., we had *a priori* assumptions that did not mandate correction for multiple comparison), we will report significant results before as well as after correction for multiple comparisons. By reporting the uncorrected *p*-values, we follow Krauth's (46) recommendation for interpreting the *p*-value and let the readers decide for themselves whether the results are significant at the significance level they may find acceptable. To correct for multiple comparisons, we used the Benjamini-Hochberg formula with the significance level *q* = 0.05 (47). Effect sizes r for the Wilcoxon Rank Sum tests were computed using the formula of Rosenthal et al. (48, 49). Hedge's g was used to compute the effect size for *t*-tests because it outperforms Cohen's d with relatively small and unbalanced group sizes as was the case with our data.

## Results

### Demographics

Table 1a shows the demographic characteristics of the three study groups at each timepoint. As expected, the two AUD groups had significantly higher scores at both timepoints than the controls for monthly drinks averaged over 1 year before treatment and over lifetime. The AUD individuals at timepoint 1 scored also significantly higher on self-reported impulsivity than the controls, whereas at timepoint 2 only the relapsers differed from the controls. Both drinking groups had significantly higher BDI scores (depressive symptomatology) than controls at timepoint 1, but only relapsers had higher BDI scores also at timepoint 2. State and trait anxiety scores were significantly higher than in controls for abstainers at timepoint 1 and timepoint 2; both scores were also significantly higher for relapsers at timepoint 1, but only the trait anxiety score remained higher than those of controls at timepoint 2.

Table 1b shows to what extent the AUD participants with three-months follow up differed from those who dropped out after timepoint 1.

### Community Configurations by Group at Timepoint 1

**Figure 2:**
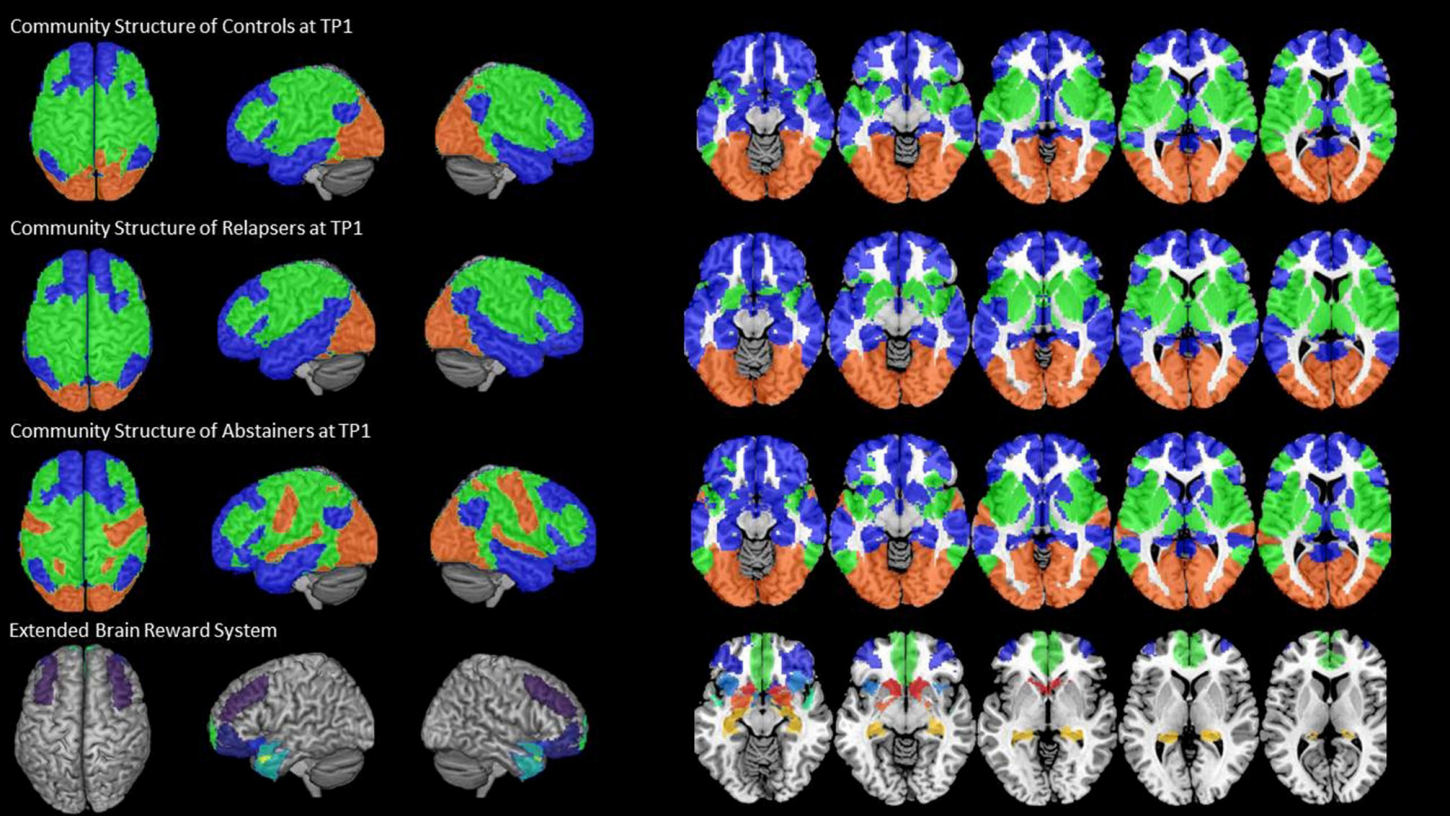
The three-community configuration and the location of the eBRS in relation to the communities. The three-community configuration of the controls (CON), the relapsers (REL), and abstainers (ABS) found at baseline are shown in the first, respectively, second and third row, the location of the eBRS within the brain is and in relation to the three communities is shown in the bottom row. The majority of the eBRS regions were allocated within community 1 (blue color).

We found an intrinsic community structure consisting of three communities in all three groups. The community structure was very similar for controls and relapsers (Figure 2, rows 1 and 2). Community 1 (blue color in Figure 2) covered bilateral superior, middle, inferior frontal and orbital regions, as well as the medial part of the prefrontal cortex, the temporal pole and the middle and inferior gyri of the temporal cortex, parts of the supramarginal and angular gyri and the cingulate cortex in the midline of the brain. Community 2 (green color in Figure 2) covered the caudal part of the frontal cortex over the motor and sensory cortex into the parietal lobe on the lateral part of the cortex also encompassing superior gyrus of the temporal cortex, parts of the insula and the rolandic operculum. Community 3 (red color in Figure 2) consisted of the occipital cortex only. The abstainers' community configuration differed from the two other groups in so far as the superior temporal gyrus, hand and face area of the motor cortex were also allocated to community 3 together with the visual cortex (Figure 2, row 3). Important in the context here is that the community allocation of the nine eBRS regions at timepoint 1 was identical for the three groups. The eBRS regions were all allocated to community 1 in all three groups except for relatively small parts of the dorsolateral prefrontal cortex located posterior to the middle frontal gyrus (Figure 2, last row). Therefore, group specific differences in the degree of the eBRS' global across-communities integration were not related to the slightly different community structure observed in controls/relapsers vs. abstainers at timepoint 1. The AUD related differences in whole-brain community configuration are the subject of separate paper.

### Analysis of the eBRS' Global Integration Coefficients (GIC)

**Figure 3:**
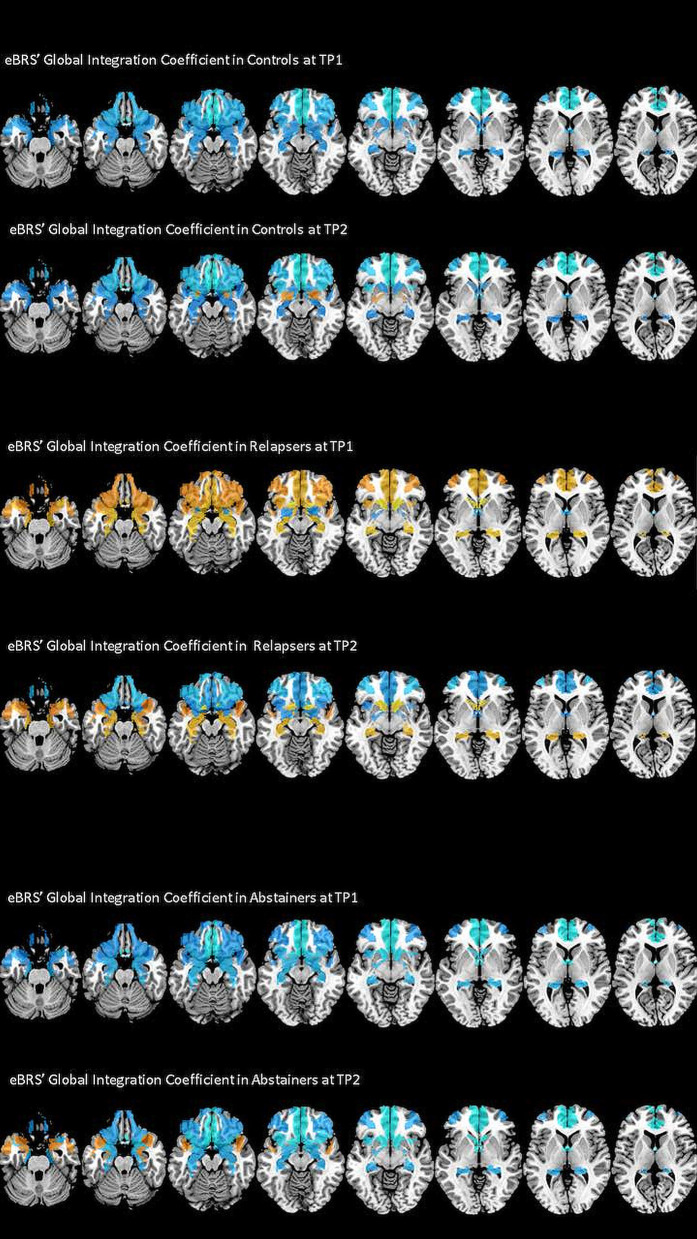
The degree of global integration of the nine eBRS regions at 1 month into abstinence ad 3 months later. eBRS regions with a positive GIC value are coded in orange – yellow (range 0–0.05) and eBRS regions with a negative GIC value are coded in blue – light blue (range 0–0.1). When compared with the controls and abstainers, relapsers showed over-integration (= positive GIC values) in almost all eBRS regions, while the eBRS in the two other groups was mostly disconnected (= negative GIC values) from the global across-community interaction.

The exact GIC mean, median and interquartile values for the three groups at both timepoints are shown in Table 3, the results of the Wilcoxon Rank Sum tests are shown in the left half of Table 4, and Figure 3 maps the GIC values on the eBRS regions for a graphical illustration of the findings. The controls' eBRS was clearly segregated from the inter-community information integration since they had negative values for all individual eBRS regions at both timepoints. Abstainers also showed negative GIC values for all of the eBRS regions, except for the dorsolateral PFC and the temporal pole at timepoint 2, indicating a general segregation of the eBRS from the inter-community-crosstalk at both timepoints. In contrast, relapsers presented with positive GIC values for six of the nine eBRS regions, the ventromedial PFC, orbital PFC, anterior insula, temporal pole, hippocampus, and nucleus accumbens at timepoint 1, which indicates a two-thirds majority of the eBRS regions was involved in the inter-community-crosstalk. However, at timepoint 2 only half these regions, the temporal pole, hippocampus and nucleus accumbens, still presented with positive GIC values in the relapsers. Table 4 shows that several of these eBRS regions showed even significant over-integration in the relapsers at both timepoints when compared with the controls, but only the *p*-value for the ventromedial PFC at timepoint 1 survived the Benjamini-Hochberg correction at timepoint 1.

**Table 3:** The global integration coefficient of the extended brain reward system (eBRS) and its constituent brain regions for the controls (CON), relapsers (REL), and abstainers (ABS).

	**Global Integration Coefficient**		
	**CON**	**REL**	**ABS**
eBRS at TP1	−0.0364/−0.0441 (0.0432)	0.0034/−0.0030 (0.0609)	−0.0551/−0.0728 (0.0959)
eBRS at TP2	−0.0412/−0.0392 (0.0792)	−0.0137/−0.0108 (0.0552)	−0.0610/−0.0714 (0.1032)
Cortical regions of the eBRS at TP1	−0.0348/−0.0489 (0.0415)	0.0283/0.0038 (0.0685)	−0.0391/−0.0409 (0.0735)
Cortical regions of the eBRS at TP2	−0.0578/−0.0677 (0.0711)	−0.0314/−0.0246 (0.0667)	−0.0461/−0.0432 (0.0528)
Dorsolateral prefrontal cortex at TP1	−0.0084/−0.0076 (0.0812)	−0.0367/−0.0392 (0.0775)	−0.0116/−0.0211 (0.1472)
Dorsolateral prefrontal cortex at TP2	−0.0252/−0.0409 (0.1429)	−0.0534/−0.0527 (0.1316)	0.0065/0.0238 (0.1254)
Ventromedial prefrontal cortex at TP1	−0.0968/−0.0992 (0.1256)	0.0219/0.0328 (0.0953)	−0.1040/−0.1101 (0.1661)
Ventromedial prefrontal cortex at TP2	−0.1219/−0.1210 (0.1358)	−0.0316/−0.0073 (0.1290)	−0.1352/−0.1019 (0.2291)
Orbital prefrontal cortex at TP1	−0.0453/−0.0364 (0.0554)	0.0059/−0.0213 (0.0979)	−0.0196/−0.0218 (0.1430)
Orbital prefrontal cortex at TP2	−0.0549/−0.0331 (0.1225)	−0.057/−0.0600 (0.0533)	−0.0304/−0.0663 (0.1224)
Anterior insula at TP1	−0.0414/−0.0471 (0.0798)	0.0165/0.0055 (0.1132)	−0.0446/−0.0276 (0.14301)
Anterior insula at TP2	−0.0717/−0.0637 (0.0641)	−0.0164/−0.0082 (0.1162)	−0.0763/−0.0607 (0.1161)
Temporal pole at TP1	−0.0271/−0.0330 (0.0787)	0.0064/−0.0180 (0.1467)	−0.01577/−0.0036 (0.1464)
Temporal pole at TP2	−0.0154/−0.0019 (0.0607)	0.0019/−0.0067 (0.0797)	0.0046/−0.0018 (0.1522)
Subcortical regions of the eBRS at TP1	−0.0272/−0.0237 (0.0590)	0.0042/0.0132 (0.0903)	−0.0751/−0.0717 (0.1272)
Subcortical regions of the eBRS at TP2	−0.0203/−0.0229 (0.0982)	0.0083/−0.0062 (0.1073)	−0.0796/−0.0935 (0.1688)
Hippocampus at TP1	−0.0240/−0.0232 (0.0755)	0.0304/0.0229 (0.0942)	−0.0507/−0.0393 (0.1266)
Hippocampus at TP2	−0.0131/−0.0006 (0.0977)	0.0242/0.0264 (0.0835)	−0.0401/−0.0601 (0.1342)
Amygdala at TP1	−0.0229/−0.0357 (0.1342)	−0.0159/−0.0392 (0.1562)	−0.0484/−0.0898 (0.1907)
Amygdala at TP2	−0.0051/−0.0017 (0.1051)	−0.0191/−0.275 (0.1206)	−0.0455/−0.0789 (0.0709)
Anterior thalamus at TP1	−0.0405/−0.0523 (0.1279)	−0.0401/−0.0549 (0.1663)	−0.1185/−0.1125 (0.1807)
Anterior thalamus at TP2	−0.0476/−0.0650 (0.1563)	−0.0113/−0.0276 (0.1791)	−0.1242/−0.1407 (0.2403)
Nucleus accumbens at TP1	−0.0215/−0.0356 (0.1435)	0.0431/0.0797 (0.1507)	−0.0825/−0.1071 (0.2492)
Nucleus accumbens at TP2	−0.0257/−0.0213 (0.1671)	0.0395/0.0265 (0.1468)	−0.1085/−0.1358 (0.2829)

*The table lists the mean, the median and the interquartil distance (in brackets) of the group-specific GIC values at each timepoint. The eBRS and its constituent brain regions were relatively over-integrated in the REL at TP1, since the REL group has generally the highest GIC values of all three groups at TP1 with exception of the GIC values of the dorsolateral prefrontal cortex*.

**Table 4:** List of significant group differences in Global Integration Coefficient (GIC) and Within Centrality of the eBRS at 1 month (TP1) into treatment and at follow-up 3 months later (TP2).

	**Global Integration Coefficient**			**Within Centrality**		
	**CON vs. REL**	**CON vs. ABS**	**REL vs. ABS**	**CON vs. REL**	**CON vs. ABS**	**REL vs. ABS**
eBRS at TP1	*p* = 0.0013 (*r* = 0.446)*		*p* = 0.0136 (r = 0.423)	*p* < 0.0001 (*g* = 1.276)*	–	*p* = 0.0002 (*g* = 1.449)*
eBRS at TP2	**–**	–	–	*p* = 0.0039 (*g* = 0.381)*	–	*p* < 0.0001 (*g* = 1.872)*
Cortical regions of the eBRS at TP1	*p* = 0.0022 (*r* = 0.425)*	–	–	*p* = 0.0175 (*g* = 0.691)	–	–
Cortical regions of the eBRS at TP2	**–**	–	–	–	–	–
Dorsolateral prefrontal cortex at TP1	**–**	–	–	–	–	–
Dorsolateral prefrontal cortex at TP2	**–**	–	–	*p* = 0.0246 (*g* = 0.763)	–	*p* = 0.0027 (*g* = 1.302)*
Ventromedial prefrontal cortex at TP1	*p* < 0.0001 (*r* = 0.571)*	–	*p* = 0.0026 (*r* = 0.532)*	*p* < 0.0001 (*g* = 1.345)*	–	*p* = 0.0003 (*g* = 1.188)*
Ventromedial prefrontal cortex at TP2	*p* = 0.0040 (*r* = 0.487)	–	*p* = 0.0242 (*r* = 0.460)	–	–	–
Orbital prefrontal cortex at TP1	*p* = 0.0485 (*r* = 0.274)	–	–	–	–	*p* = 0.0409 (*g* = 0.776)
Orbital prefrontal cortex at TP2	**–**	–	–	–	–	–
Anterior insula at TP1	*p* = 0.0201 (*r* = 0.322)	–	–	–	–	–
Anterior insula at TP2	*p* = 0.0451 (*r* = 0.339)	–	–	–	–	–
Temporal pole at TP1	**–**	–	–	–	–	–
Temporal pole at TP2	**–**	–	–	–	–	*p* = 0.0269 (*g* = 1.079)
Subcortical regions of the eBRS at TP1	**–**	–	*p* = 0.009 (*r* = 0.448)	*p* < 0.0001 (*g* = 1.197)*	–	*p* < 0.0001 (*g* = 1.578)*
Subcortical regions of the eBRS at TP2	**–**	–	*p* = 0.0207 (*r* = 0.472)	*p* = 0.0014 (*g* = 1.141)*	*p* = 0.0083 (*g* = 1.086)*	*p* < 0.0001 (*g* =2.307)*
Hippocampus at TP1	*p* = 0.0103 (*r* = 0.356)	–	*p* = 0.0136 (*r* = 0.423)	–	–	–
Hippocampus at TP2	**–**	–	–	–	*p* = 0.0054 (*g* = 1.075)*	*p* = 0.0027 (*g* = 1.321)*
Amygdala at TP1	**–**	–	–	–	–	–
Amygdala at TP2	**–**	–	–	–	–	*p* = 0.0278 (*g* = 0.936)
Anterior thalamus at TP1	**–**	–	–	–	–	–
Anterior thalamus at TP2	**–**	–	–	*p* = 0.0215 (*g* = 0.778)	–	*p* = 0.0494 (*g* = 0.753)
Nucleus accumbens at TP1	*p* = 0.0109 (*r* = 0.353)	–	*p* = 0.0065 (*r* = 0.467)*	*p* < 0.0001 (*g* = 1.521)*	–	*p* < 0.0001 (*g* = 1.755)*
Nucleus accumbens at TP2	**–**	–	*p* = 0.0242 (*r* = 0.458)	*p* < 0.0001 (*g* = 1.578)*	*p* = 0.004 (*g* = 1.191)*	*p* < 0.0001 (*g* = 2.747)*

*The results of the Wilcoxon rank sum tests used to test for group differences in the not-normally distributed GIC values and the corresponding effects sizes r (in brackets) computed following the formula of Rosenthal et al. (48) are listed on the left side of the table. The results of the Student t-tests used to test for group differences in the normally distributed Within Centrality and the corresponding effect sizes (Hodge's g) are listed on the right side of the table. Asteriks indicate p values that stay significant at q = 0.05 after Benjamini-Hochberg correction for multiple comparisons*.

To investigate which one of the two other communities contributed to the ventromedial PFC's over-integration in the relapsers, we computed a Spearman correlation between the average GIC values of the communities with the GIC values of the ventromedial PFC. Table 5 shows that the GIC values of the ventromedial PCF - as expected - were significantly positively correlated with its own community 1 in all three groups at both timepoints. However, the ventromedial PFC was negatively associated with the other two communities in the controls and abstainers, although these negative correlations reached statistical significance only in the abstainers for both communities at timepoint 1. In contrast to the two other groups, the GIC values of the relapsers' ventromedial PFC at timepoint 1 showed significant positive association with community 3 but not with community 2. This suggests an over-integration of the ventromedial PFC with the (occipital) community 3 in the relapsers at timepoint 1. At timepoint 2, the controls and the relapsers showed the expected association patterns – a significant positive correlation of the ventromedial PFC's GIC values with its own community– and essentially no associations with communities 2 and 3.

**Table 5:** Results of the *post-hoc* tests – Over-Integration of the ventromedial PFC in relation to the three community-structure.

		**Ventromedial Prefrontal Cortex**		
		**Community 1**	**Community 2**	**Community 3**
TP 1	CON	ρ = 0.687; *p* < 0.0001**	ρ = −0.231; *p* = 0.220	ρ = −0.226; *p* = 0.229
	REL	ρ = 0.636; *p* = 0.0015*	ρ = −0.135 *p* = 0.563	ρ = 0.476 *p* = 0.0251*
	ABS	ρ = 0.608; *p* = 0.036*	ρ = −0.664; *p* = 0.018*	ρ = −0.622; *p* = 0.031*
TP 2	CON	ρ = 0.735; *p* = 0.0001**	ρ = −0.129; *p* = 0.579	ρ = −0.036; *p* = 0.876
	REL	ρ = 0.737; *p* = 0.0027*	ρ = −0.055; *p* = 0.852	ρ = 0.007; *p* = 0.982
	ABS	ρ = −0.988; *p* = < 0.0001**	ρ = −0.515; *p* = 0.128	ρ = −0.551 *p* = 0.098

*Significant Spearman's ρ correlations are highlighted with one asterisk for the significance level <0.05 and with two asterisks for the significance level <0.001. The ventromedial PFC in the CON was negatively but not significantly correlated with communities 2 and 3 at both timepoints (TP). REL showed significant positive correlations for ventromedial PFC and community 3 at timepoint 1, indicating over-integration, but not anymore at timepoint 2. The ventromedial PFC in the ABS was negatively and significantly correlated with communities 2 at timepoint 1 but not anymore at timepoint 1*.

### Within-Community Centrality

**Figure 4:**
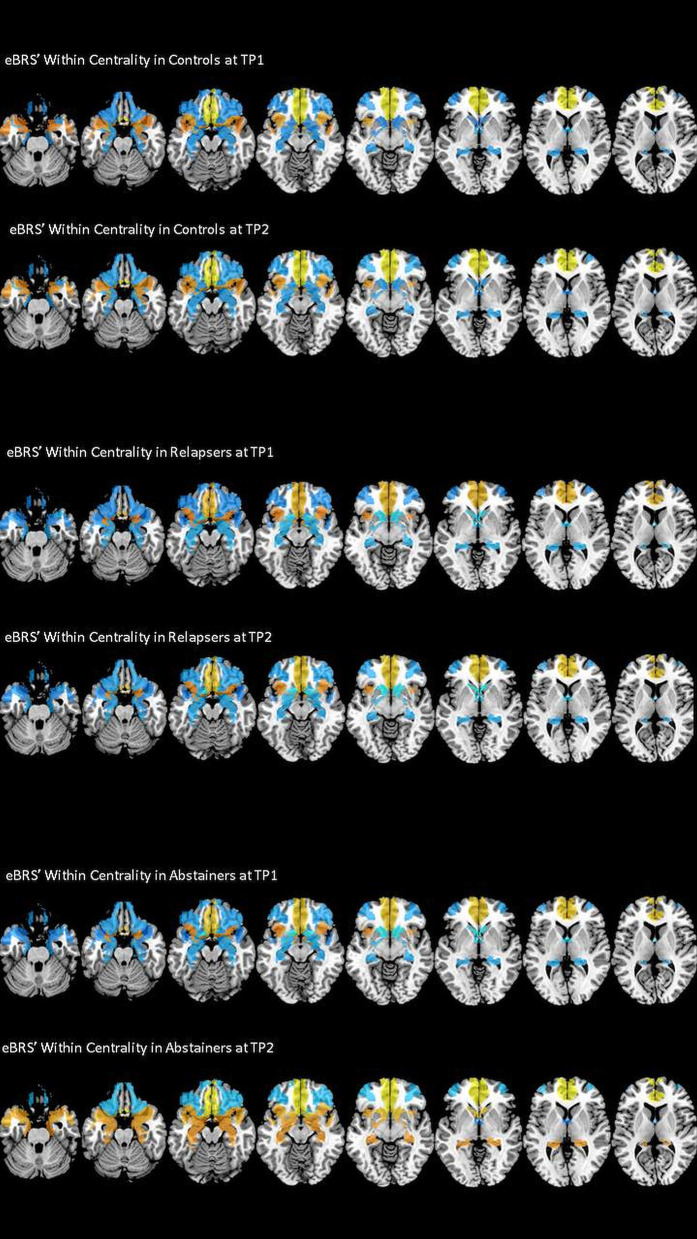
Within Centrality of the nine eBRS regions at 1 month into abstinence ad 3 months later. eBRS regions with a positive Within Centrality value are coded in orange – yellow (range 0–0.8) and eBRS regions with a negative Within Centrality value are coded in blue – light blue (range 0–1). Relapsers had generally lower Within Centrality values than the two other groups at both timepoints, abstainers showed significantly higher Within Centrality values in subcortical regions, especially in the hippocampus and nucleus accumbens, than controls and relapsers at the 3 months follow-up.

We found that the ventromedial PFC had the highest within-community centrality values in all three groups at both timepoints and together with the anterior insula it qualified as a provincial hub in all three groups (see Table 6 for the exact mean and standard deviation values, right half of Table 4 for the results of the *t*-tests, and Figure 4 maps the within-community centrality values on the eBRS region for a graphical illustration of the findings). Three other features seem noteworthy: firstly, and as expected, the within-community configuration of the controls (with ventromedial PFC, anterior insula and temporal pole all qualifying as provincial hubs) was stable across the two timepoints. Secondly, besides the anterior insula and ventromedial PFC, none of the eBRS regions in the relapsers qualified as a provincial hub at timepoint 1. Additionally, with exception of two frontal eBRS regions (the dorsolateral PFC and the orbital PFC, which both do not belong to the core BRS but are considered part of the extended BRS), relapsers also had always the lowest within-community centrality values of all three groups, with the differences for the ventromedial PFC and nucleus accumbens at timepoint 1 statistically significant when compared with the controls and surviving the subsequent Benjamini-Hochberg correction. Three months later, after the relapsers had resumed alcohol consumption, they still had globally lower within-centrality values than the controls with the anterior thalamus, the nucleus accumbens, and additionally the dorsolateral PFC being significantly lower, but only the *p*-value of nucleus accumbens also surviving the correction for multiple comparisons. Thirdly, abstainers at both timepoints had always the highest within-centrality values of the three groups with exception of the dorsolateral PFC and the orbital PFC, for which they had the lowest within-centrality values at both times. As a result, abstainers had more eBRS regions qualifying as provincial hubs than controls and relapsers at both timepoints. At timepoint 1, the ventromedial PFC, anterior insula, temporal pole, and the anterior thalamus qualified as provincial hubs. Three months later, the number of provincial hubs in the abstainers' eBRS was even higher with the ventromedial PFC, anterior insula, temporal pole, nucleus accumbens, amygdala, and hippocampus qualifying as provincial hubs at timepoint 2. The subsequent *t*-tests for differences in within-community-centrality between controls and abstainers at timepoint 2 showed significant group differences for the hippocampus and nucleus accumbens that survived the correction for multiple comparisons.

**Table 6:** The within centrality of the extended brain reward system (eBRS) and its constituent brain regions in the controls (CON), relapsers (REL), and abstainers (ABS) at 1 month (TP1) into treatment and at follow-up 3 months later (TP2).

	**Within Centrality**		
	**CON**	**REL**	**ABS**
eBRS at TP1	0.0049 (0.2003)	−0.2436 (0.1778)	0.0359 (0.2017)
eBRS at TP2	−0.1523 (0.2194)	−0.2391 (0.2397)	0.1471 (0.1448)
Cortical regions of the eBRS at TP1	0.1733 (0.1843)	0.0290 (0.2384)	0.1341 (0.2176)
Cortical regions of the eBRS at TP2	0.1473 (0.1743)	0.1022 (0.2610)	0.1171 (0.1293)
Dorsolateral prefrontal cortex at TP1	−0.1114 (0.4122)	−0.0794 (0.5655)	−0.3486 (0.5492)
Dorsolateral prefrontal cortex at TP2	−0.1067 (0.4851)	0.2491 (0.3454)	−0.3347 (0.4669)
Ventromedial prefrontal cortex at TP1	0.8596 (0.2856)	0.3953 (0.4138)	0.8757 (0.3856)
Ventromedial prefrontal cortex at TP2	0.7934 (0.3303)	0.5350 (0.4612)	0.8445 (0.5252)
Orbital prefrontal cortex at TP1	−0.1600 (0.4266)	−0.1255 (0.3833)	−0.4341 (0.4233)
Orbital prefrontal cortex at TP2	−0.3087 (0.3559)	−0.3080 (0.5440)	−0.5214 (0.3037)
Anterior insula at TP1	0.2710 (0.4351)	0.0259 (0.5111)	0.3529 (0.5579)
Anterior insula at TP2	0.2173 (0.5129)	0.0805 (0.5947)	0.2776 (0.3284)
Temporal pole at TP1	0.0072 (0.4142)	−0.0704 (0.4172)	0.2247 (0.4153)
Temporal pole at TP2	0.1414 (0.4298)	−0.0455 (0.3643)	0.3197 (0.2966)
Subcortical regions of the eBRS at TP1	−0.2054 (0.3359)	−0.5843 (0.2875)	−0.0868 (0.3452)
Subcortical regions of the eBRS at TP2	−0.2185 (0.3901)	−0.6659 (0.3955)	0.1845 (0.3250)
Hippocampus at TP1	−0.2466 (0.3918)	−0.4128 (0.5059)	−0.1500 (0.5297)
Hippocampus at TP2	−0.2821 (0.3681)	−0.3535 (0.3253)	0.1320 (0.4208)
Amygdala at TP1	−0.1762 (0.5847)	−0.4825 (0.6132)	−0.1466 (0.5626)
Amygdala at TP2	−0.2966 (0.6699)	−0.4582 (0.7446)	0.1793 (0.5773)
Anterior thalamus at TP1	−0.3836 (0.6392)	−0.6222 (0.7669)	0.2164 (0.5956)
Anterior thalamus at TP2	−0.0951 (0.6114)	−0.6823 (0.9323)	−0.0853 (0.5315)
Nucleus accumbens at TP1	−0.0151 (0.5071)	−0.8199 (0.6019)	−0.2672 (0.6515)
Nucleus accumbens at TP2	−0.2003 (0.6036)	−1.1694 (0.6302)	0.5119 (0.5847)

*The table lists the mean and the standard deviation (in brackets) of the normally distributed group-specific within centrality values at each timepoint. With exception of the dorsolateral prefrontal cortex and the orbital prefrontal cortex, abstainers had generally the highest Within Centrality values of all three groups, whereas relapsers had the smallest Within Centrality values*.

## Discussion

The aim of our rs-fMRI analyses was to investigate the degree to which relapse or abstinence in AUD treatment seekers affects the brain's intrinsic community structure. In particular, we hypothesized that 1. the interaction of the eBRS within its own community is differently altered in relapsers and abstainers when compared with control participants, and 2. the interaction of the eBRS regions with the other communities (between-community crosstalk) differs between relapsers and abstainers when compared with controls. In the subsequent sections we discuss to what extent our findings supported our two hypotheses and lead up to our main conclusions.

### Within-Community Analysis

Our fist hypothesis that the interaction of the eBRS within its own community is differently altered in relapsers and abstainers when compared with control participants was confirmed. Analyzing the role of the eBRS regions and the whole eBRS within their own community revealed significant differences between abstainers, relapsers, and controls at timepoint 1, which were even more accentuated at timepoint 2. The controls had a middle position between the two AUD groups with the AUD groups showing an opposite pattern since abstainers had generally the highest within-centrality values when relapsers had generally the lowest within-centrality values or vice versa. Only the ventromedial PFC and anterior insula qualified as provincial hubs in all three groups at both timepoints, suggesting that these two eBRS regions were central for the within-community crosstalk regardless of group membership or time. While the within-community configuration of the controls was stable across the two timepoints, the abstainers underwent a prominent within-community re-configuration. As a result, they had two times more eBRS regions qualifying as provincial hubs than the controls at timepoint 2. Additionally, while the eBRS regions' within-community configuration between controls and abstainers was not significantly different at timepoint 1, 3 months later, hippocampus and nucleus accumbens had qualified as new additional provincial hubs in the abstainers but both regions had also significant higher within-centrality surviving the Benjamini-Hochberg correction. In contrast to the abstainers, relapsers stayed relatively stable with the exception of the dorsolateral PFC showing an increase in within-centrality to the effect that this region newly qualified as a provincial hub at timepoint 2. Since this eBRS region had consistently negative within-centrality values in controls and abstainers (and qualified in the abstainers even as a hub for between-community crosstalk at timepoint 2) and the dorsolateral PFC's prominent role in executive control (50), this change in the relapsers might be rather an indicator for a maladaptive than beneficial process.

### Across-Community Analysis

Our second hypothesis pertained to the eBRS regions' global crosstalk with other communities, specifically that we would find significant differences between the three groups. The eBRS regions of controls and abstainers showed the expected negative GIC values, reflecting relative segregation from the inter-community-communication at both timepoints. In contrast, relapsers had generally higher GIC values than the other two groups and even positive GIC values in six of the nine eBRS regions (with the ventromedial PFC surviving multiple comparison corrections) at timepoint 1, which shows that these eBRS regions were engaged in the global information integration across communities early in treatment. The subsequent *post hoc* correlation analyses showed a positive association of the ventromedial PFC in relapsers with community 3, which was practically identical with the occipital cortex. This positive association is all the more remarkable as the ventromedial PFC is a core region of the default mode network (DMN), the most salient feature of which is its anticorrelated activity with task-positive networks like the visual network. Controls and abstainers precisely showed that negative association, equivalent with segregation of the ventromedial PFC and the visual cortex in these two groups during rest.

The meaning of the brain's intrinsic brain activity and how it generates these patterns of dynamic syn- and desynchronization is still not fully understood. However, it has been shown that this intrinsic activity is also present during active task-performance (43), and that it shapes the functional network architecture during tasks (51). Consequently, the finding that the ventromedial PFC of the relapsers seemed to be involved in global information integration with the visual cortex already during rest may suggest that the relapsers have something like an intrinsic processing advantage whenever it is necessary that the ventromedial PFC and - by virtue of the ventromedial PFC's role as the most prominent provincial hub within the eBRS – the other eBRS regions have to actively interact with regions of the visual cortex.

However, there is evidence that this intrinsic over-connectivity of the ventromedial PFC with the visual cortex found in the relapsers is not at all a processing advantage but may rather make them more vulnerable to relapse. It has repeatedly been shown that AUD individuals have a higher degree of brain activation in the medial PFC, the orbitofrontal cortex, anterior cingulate cortex, insula, and striatum in response to alcohol cues (52, 53), and that this increased activation predicts relapse in detoxified AUD individuals (54). Also, the primary and secondary visual cortices respond with significant higher activation when confronted with alcohol vs. neutral cues (53). Therefore, stronger interactions between visual cortex and ventromedial PFC, together with structural alterations in the prefrontal cortex generally reported in AUD individuals such as our study participants that may interfere with top-down control (55–59), and put an individual at greater risk of relapse.

That relapsers might have something like a maladaptive processing advantage–particularly for alcohol-related cues – was also demonstrated by EEG (60). The N170 is an event related potential reflecting a very early, bottom-up, and subconscious process in the visual cortex. It is commonly associated with visual face processing but has also been found for stimuli that related to other kinds of expertise knowledge (61, 62). Compared with abstainers, relapsers showed a significantly higher N170 amplitude in a Go-NoGo experimental setting when instructed to focus attention on the neutral cue and to ignore the alcohol-related cue (60). That increased N170 was specific for the condition in which the alcohol-related cue had to be ignored. When the cue was presented with a neutral beverage cue (i.e., a picture of a pitcher and a glass with orange juice instead of a picture with a beer bottle and a full glass of beer), the N170 amplitude did not differ between relapsers and abstainers (60). Based on these findings the authors concluded that a heightened N170 amplitude in response to a to-be-ignored alcohol cue is specifically related to a high relapse risk.

Another aspect of the medial PFC-visual cortex over-integration pattern found in treatment-seekers while they are still abstinent corroborates the clinical relevance. The ventromedial PFC is a core region of the DMN, and it was – together with the other eight eBRS regions – consistently allocated to community 1 in the relapsers. One of the most striking features of this community was how much it resembled the DMN in its anatomical topography. Our finding that the DMN interacts with the visual cortex in AUD individuals is supported by another study, Fede et al. (63) used a machine learning prediction analysis and found that the connectivity between the DMN and the visual networks predicted reliably the AUD severity (measured by AUDIT scores) in adults with problematic drinking patterns. While our two AUD groups did not significantly differ in AUD severity, the stronger crosstalk between DMN and visual networks (i.e., their over-integration) at 1 month of abstinence while in treatment was associated with a negative treatment outcome within the subsequent 3 months.

### Study Limitations

One limitation of the study is certainly the unbalanced group sizes and relatively small sample size of the abstainers at both timepoints. We had only 34 AUD individuals with acceptable data for timepoint 1, and only 24 of them had also acceptable data for the second timepoint. The reason for the relatively modest sample size is 2-fold: One, the rate of relapse within the first 6 months after treatment is substantial, so that we had many more relapsers than abstainers in our study, and two, motion artifacts can seriously compromise the data quality of all types of fMRI analyses, because even small head movements can falsely increase BOLD signal correlations. To assure the high quality of our analyses, we therefore employed rigorous censoring and denoising procedures and only used rs-fMRI data of participants with at least 5 min of data. Although all three groups had a similar proportion of data excluded for excessive motion or other noise, the abstainer sample was already small to start with.

We found characteristic differences in intrinsic network organization in future relapsers and successful abstainers early in treatment. However, we do not know whether these two groups did already differ in their intrinsic network organization when starting the treatment or whether the differences resulted from different neuroplastic alterations during the 1st month of sobriety. We also are not able to say whether these intrinsic network organization differences are not just the result of different rates for neuroplastic re-configuration over time, resulting in potentially similar configurations had the relapsers been able to stay sober for a longer time.

### Conclusion

This study focussed on how the eBRS interacts with the regions within its own community and with the other intrinsic communities of the brain. It showed that the eBRS of the abstainers was not significantly different from that of the controls in the degree of across-community-crosstalk at either timepoint; however, the abstainers demonstrated a significant re-configuration of the eBRS regions' involvement in the crosstalk within their own community 1 month into treatment in so far as that the eBRS had a far more central role within its own community than it had in the other two groups. This re-configuration was even more pronounced 3 months later during abstinence. In contrast, the relapsers 1 month into treatment showed an over-integration of most of the eBRS regions when compared with the controls, especially a significant over-integration of the ventromedial PFC with the visual cortex while still abstinent. We suggest that this over-integration contributed to the relapsers' subsequent inability to remain abstinent and, as such, is an early indicator of subsequent treatment failure. Three months later after having relapsed to heavy alcohol consumption, the relapsers' community configuration was no longer over-integrated relative to that of the controls, and the relapsers (mal)functioned again in regard to substance abuse as they had before treatment.

## Data Availability Statement

The datasets for this article are not publicly available because they were created as part of Veteran's Administration approved research. Requests to access the datasets should be directed to the senior author (dieter.meyerhoff@ucsf.edu).

## Ethics Statement

The studies involving human participants were reviewed and approved by Human Research Protection Program IRB; (Formerly CHR) University of California San Francisco and SF VA Medical Center (SF VAMC). The patients/participants provided their written informed consent to participate in this study.

## Author Contributions

DM and AM contributed conception, design of the study, contributed to manuscript revision, read, and approved the submitted version. AM performed the analyses and wrote the first draft of the manuscript.

## Conflict of Interest

The authors declare that the research was conducted in the absence of any commercial or financial relationships that could be construed as a potential conflict of interest.
